# Functions of Smartphone Apps and Wearable Devices Promoting Physical Activity: Six-Month Longitudinal Study on Japanese-Speaking Adults

**DOI:** 10.2196/59708

**Published:** 2024-12-10

**Authors:** Naoki Konishi, Takeyuki Oba, Keisuke Takano, Kentaro Katahira, Kenta Kimura

**Affiliations:** 1 Human Informatics and Interaction Research Institute The National Institute of Advanced Industrial Science and Technology (AIST) Tsukuba, Ibaraki Japan

**Keywords:** mHealth, mobile health, smartphone app, physical activity, wearable activity tracker, longitudinal design, wearable, Japan, health promotion

## Abstract

**Background:**

Smartphone apps and wearable activity trackers are increasingly recognized for their potential to promote physical activity (PA). While studies suggest that the use of commercial mobile health tools is associated with higher PA levels, most existing evidence is cross-sectional, leaving a gap in longitudinal data.

**Objective:**

This study aims to identify app-use patterns that are prospectively associated with increases in and maintenance of PA. The primary objective was to test whether continued app use is linked to adherence to the recommended PA levels (ie, 23 metabolic equivalent task [MET] hours per week for adults or 10 MET hours/week for individuals aged >65 years) during a follow-up assessment. The secondary objective was to explore which functions and features of PA apps predict changes in PA levels.

**Methods:**

A 2-wave longitudinal survey was conducted, with baseline and follow-up assessments separated by 6 months. A total of 20,573 Japanese-speaking online respondents participated in the baseline survey, and 16,286 (8289 women; mean age 54.7 years, SD 16.8 years) completed the follow-up. At both time points, participants reported their current PA levels and whether they were using any PA apps or wearables. Each participant was classified into 1 of the following 4 categories: continued users (those using apps at both the baseline and follow-up; n=2150, 13.20%), new users (those who started using apps before the follow-up; n=1462, 8.98%), discontinued users (those who had used apps at baseline but not at follow-up; n=1899, 11.66%), and continued nonusers (those who had never used apps; n=10,775, 66.16%).

**Results:**

The majority of continued users (1538/2150, 71.53%) either improved or maintained their PA at the recommended levels over 6 months. By contrast, discontinued users experienced the largest reduction in PA (−7.95 MET hours/week on average), with more than half failing to meet the recommended levels at the follow-up (n=968, 50.97%). Analyses of individual app functions revealed that both energy analysis (eg, app calculation of daily energy expenditure) and journaling (eg, users manually entering notes and maintaining an exercise diary) were significantly associated with increases in PA. Specifically, energy analysis was associated with an odds ratio (OR) of 1.67 (95% CI 1.05-2.64, P=.03), and journaling had an OR of 1.76 (95% CI 1.12-2.76, P=.01). By contrast, individuals who maintained the recommended PA levels at the follow-up were more likely to use the goal setting (OR 1.73, 95% CI 1.21-2.48, P=.003), sleep information (OR 1.66, 95% CI 1.03-2.68, P=.04), and blood pressure recording (OR 2.05, 95% CI 1.10-3.83, P=.02) functions.

**Conclusions:**

The results highlight the importance of continued app use in both increasing and maintaining PA levels. Different app functions may contribute to these outcomes, with features such as goal setting and journaling playing a key role in increasing PA, while functions related to overall health, such as sleep tracking and blood pressure monitoring, are more associated with maintaining high PA levels.

## Introduction

### Background

Physical inactivity is highly prevalent in modern society, with an average global prevalence of 31% [1]. It is associated with noncommunicable diseases such as stroke, hypertension, and diabetes [2] and is now the fourth leading risk factor for mortality [3]. To promote physical activity (PA), behavior change techniques (BCTs) have been developed [4], which can be delivered either in-person (eg, by health care professionals) or digitally (eg, through smartphones and wearable activity trackers). These digital mobile health (mHealth) interventions are expected to play a pivotal role in promoting PA, particularly during and after the COVID-19 pandemic, when in-person contact is highly restricted. These digital PA tools typically offer measurement and monitoring (eg, activity logs), information and analysis (eg, progress and individual exercise data), and support and feedback (eg, advice on PA and goal setting) [5]. The most frequently implemented BCTs include self-monitoring, providing feedback on performance, and goal-setting [6]. Observational studies have found that fitness app users are more physically active than nonusers [7], with app users having approximately twice the odds of meeting aerobic PA guidelines compared with nonusers, even during the COVID-19 pandemic [8]. One important limitation in the literature is that most studies on PA apps (and health care apps in general) have used a cross-sectional design, meaning longitudinal evidence is still lacking. We aimed to fill this gap and investigate whether, and how, daily use of PA apps and wearables (not necessarily as part of a clinical intervention or treatment) is prospectively associated with increased levels of PA.

### How Effective Is an mHealth Intervention? Evidence From Clinical Trials

A large number of randomized and nonrandomized trials have been published on this topic, not limited to daily use of commercial PA apps and wearables. To our knowledge, 3 umbrella reviews have been conducted, focusing on digital interventions for improving PA. An early umbrella review [9] synthesized 11 systematic reviews and meta-analyses on eHealth or mHealth interventions targeting PA, sedentary behavior, and healthy eating for healthy individuals. The authors concluded that the majority of eHealth/mHealth interventions were reported as effective, although high heterogeneity was observed across multiple studies. Another umbrella review [10] focused on interventions using wearable activity trackers to improve PA. A synthesis of 39 systematic reviews and meta-analyses indicated a moderate effect size (standardized mean difference 0.3-0.6). A more recent umbrella review [11] identified 17 systematic reviews and meta-analyses on digital interventions specifically targeting PA and sedentary behavior to prevent or manage noncommunicable diseases. The results suggest that digital interventions have a small to moderate effect on increasing PA, although heterogeneity is documented across multiple reviews. For example, 3 systematic reviews concluded that mHealth interventions are effective, particularly those involving gamification [12], personalization [13], or delivery in workplace settings [14]. By contrast, a meta-analysis [15] found no significant effects of mobile interventions on total PA, moderate to vigorous PA, or walking. Similarly, a review of mHealth interventions equipped with social features found a nonsignificant effect on PA outcomes [16].

Researchers have also explored specific components or features of apps and wearables that are key to improving PA. Apps and digital interventions offering richer content and a larger number of BCTs are found to be more favored by users [17] and are associated with better health outcomes [18,19], although users also appreciate simplicity (eg, ease of use) [20]. Researchers have also found that certain app features and characteristics are more favored than others, such as data export, usability, and cost [20]; tracking (eg, steps, heart rate, and ovulation) [21,22]; and health information and medical reminders [23]. However, the umbrella reviews [9,11] concluded that the evidence for the effectiveness of specific BCTs or combinations of BCTs in digital PA interventions is largely mixed. In the search for successful digital implementations of BCTs, meta-regressions and systematic reviews highlighted the importance of behavioral goals and self-monitoring [24]; SMS text messaging, personalization, goal setting and planning, and graded tasks [25]; goal setting, prompts/cues, feedback on behavior, and action planning [26]; and personalized goal setting with motivational feedback [27]. By contrast, several meta-analyses reported no significant associations between intervention efficacy and the number or types of BCTs implemented [28,29].

Note that these analyses typically targeted clinical trials focused on specific populations, such as patients, older adults, and individuals with low socioeconomic status. Only a few studies have investigated how the spontaneous use of commercial apps and wearables can help improve PA in a community sample. Investigating app use in uncontrolled settings is particularly important to assess the potential efficacy of PA apps and wearables on the market. Studies have highlighted substantial differences in user behavior between controlled clinical contexts and real-world settings. For example, the average retention rate of mHealth interventions in published randomized controlled trials is about 91% [25], which is surprisingly high compared with the user engagement observed with commercial health care apps (eg, 4%, the median percentage of daily active users of mental health apps) [30]. An exceptional longitudinal study [31] investigated user engagement with a commercial app that rewards users with digital incentives for walking. The results showed that 60% of participants engaged with the app for at least 6 months. Interestingly, users who actively engaged with the app experienced larger increases in daily step count compared with less frequent users. These findings highlight the importance of continuous, long-term use of PA apps or wearables for users to fully benefit from these digital tools.

### Study Overview

The general purpose of this study was to identify app-use patterns prospectively associated with increased levels of PA. To achieve this, we conducted a 2-wave longitudinal survey with a 6-month interval. At each survey point, participants reported their current levels of PA and whether they were using any PA apps or wearables. Each participant was classified into 1 of 4 categories: continued users (those who used apps or wearables at both baseline and follow-up), new users (those who started using apps before the follow-up), discontinued users (those who used apps at baseline but not at follow-up), and continued nonusers (those who had never used apps). We were particularly interested in (1) whether continued and new users would increase their PA levels or maintain high-level PA at the 6-month follow-up, and (2) whether any features of the PA apps would predict increases in PA.

Our recent cross-sectional study found that users typically engaged with a limited number of functions within an app (median 2 functions, IQR 1-4 functions). Physically active users tended to use functions such as sensor information (eg, step count and heart rate), goal setting (eg, setting a daily step goal), energy analysis (eg, estimating calories burned), journaling (eg, manually recording daily exercise), and global positioning system (GPS)/maps. We did not have a specific hypothesis regarding the prospective effects of individual app functions, so the overall analyses were conducted in an exploratory manner. However, we expected that sensor information (closely related to self-monitoring/tracking in the BCT taxonomy) and other functions implementing regulatory techniques [32] would be associated with increases in PA. Additionally, we expected that the number of functions might be linked to PA increases, as some studies have suggested that the amount of app content or the number of implemented BCTs is associated with the efficacy of mobile interventions [18,19].

### Objectives

In this study, we had 2 objectives: the primary objective was to investigate whether new and continued users would increase PA or maintain high levels of PA over 6 months. The secondary objective was to identify the functions and features of PA apps that would predict increases in and maintenance of PA.

## Methods

### Participants and Procedure

Participants were recruited from the respondents who completed the first (baseline) survey, the results of which have been published elsewhere [33,34]. The baseline survey was conducted in 2023. Invitations to participate were sent to potential participants (residents of Japan who were registered in a database for online surveys) [33,34]. At baseline, 20,573 online respondents completed questionnaires regarding general health and health-related behaviors, including PA levels and use of mHealth apps. The only eligibility criterion was age (>18 years), with no additional criteria. Proficiency in Japanese was assumed, as the survey was written in Japanese. All participants were invited to the second (follow-up) survey, which took place approximately 6 months after the baseline survey. The follow-up survey, which included the same questions on PA and mHealth use, was completed by 16,286 of the 20,573 participants. Data from those who completed the follow-up were used for statistical analyses. Because of the online nature of the surveys, the reasons for dropout could not be tracked. Specifically, we had no means of reminding participants about the follow-up survey other than via email. Tracking dropouts was technically impossible, as they no longer responded to our emails. Participants received a small compensation for each survey (an online shopping voucher valued at approximately US $0.31).

### Ethics Approval

The study protocol was approved by the Ethics Committee of the National Institute of Advanced Industrial Science and Technology (approval ID 2022-1279).

### Measures

#### International Physical Activity Questionnaire-Short Form

At each survey, participants reported how many days and minutes per day they engaged in (1) walking, (2) moderate-intensity activity, and (3) vigorous-intensity activity over an average week [35,36]. The reported duration and frequency of each activity were then multiplied and converted into metabolic equivalent task (MET) hours per week. To determine whether each participant had a sufficient level of PA, the MET score was dichotomized to represent adherence to the national PA criteria recommended by the Ministry of Health, Labour and Welfare of Japan: 23 MET hours/week for adults aged <65 years and 10 MET hours/week for older adults [37]. Although there has been debate about dichotomization, we chose this approach because using an established cutoff helps clarify whether each participant achieved a clinically meaningful level of PA. Another advantage is that it explicitly distinguished those who maintained low or high levels of PA over time, which could not be separated using a simple numeric change score.

#### Stages of Change Questionnaire

Participants completed the Japanese version [38] of the Stages of Change questionnaire for PA, based on the transtheoretical model [39,40]. The questionnaire asked participants to select the most applicable statement from the following 5 statements: *I currently do not exercise and do not intend to start exercising in the future* (Precontemplation); *I currently do not exercise but I am thinking about starting to exercise in the next 6 months* (Contemplation); *I currently exercise some, but not regularly* (Preparation); *I currently exercise regularly, but have only begun doing so within the last 6 months* (Action); and *I currently exercise regularly and have done so for longer than 6 months* (Maintenance). Regular exercise was explicitly defined in the questionnaire instructions as engaging in PA for at least 20 minutes, twice or more per week.

### Use of Apps and Wearables

At baseline, participants provided a binary response indicating whether they used any apps or wearables to support their PA and exercise. Those who answered affirmatively were then asked for details on how they used the apps and wearables. The questions included the duration of app use, with the following response options: <1 week, <1 month, <3 months, <6 months, <1 year, and ≥1 year. Participants were also asked about the functions and features of the apps they were using. They were presented with a list of 41 app functions (eg, sensor information, goal setting, and energy analysis) [33] and indicated any that applied to their usage [20,29]. However, as most of the listed functions were rarely used [33], we focused exclusively on the most frequently used functions for the current analysis: sensor information (eg, step count and heart rate), goal setting and progress tracking (eg, steps achieved), energy analysis (eg, estimated daily energy expenditure), weight recording, journaling (eg, manually entered diary or notes), GPS/maps, sleep information, reward points, and blood pressure recording.

At the 6-month follow-up, participants completed a similar questionnaire asking whether they were using apps and wearables. Unlike at baseline, participants were given 3 response options: (1) have been using apps and wearables for the past 6 months, (2) used them previously but no longer, and (3) have never used any app or wearable. Participants were also asked about the duration and frequency of app use (ie, how long and how often they had used/been using the app). However, questions regarding individual app functions and features were omitted due to limited space in the follow-up survey.

**Figure 1:**
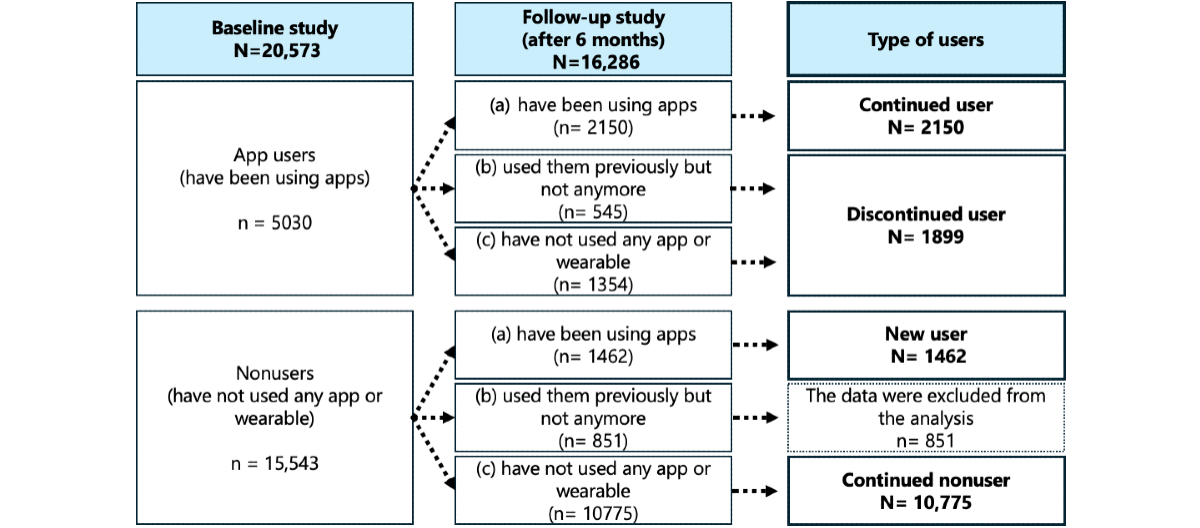
Overview of user type classification.

Responses from baseline and follow-up were interpreted as a 2×2 factorial matrix (user vs nonuser; baseline vs follow-up), classifying each participant into 4 categories (Figure 1): new users (those who began using apps before the follow-up), continued users (those who used apps or wearables at both baseline and follow-up), discontinued users (those who used apps at baseline but not at follow-up), and continued nonusers (those who never used apps). Participants who indicated at follow-up that they had used apps or wearables but were no longer using them (ie, those who selected option 2) were classified as discontinued users if they were identified as app users at baseline. If they were identified as nonusers at baseline, they were excluded from the statistical analyses for ease of interpretation. These participants were considered temporary app users—they may have used apps for a short period between baseline and follow-up but did not show significant changes in PA levels (*P*=.29; see Figure S1 in Multimedia Appendix 1 for details). We did not consider their usage comparable to that of the other participant groups.

### Statistical Analyses

First, we explored demographic and descriptive differences between the 4 types of users based on gender, age, education level, household income, PA level, and readiness. Second, logistic regression analyses were conducted to examine how continued and discontinued app usage are associated with changes in PA levels. Two binary dependent variables were used to represent the following contrasts: (1) individuals who maintained underrecommended levels of PA (<23 or 10 METs) at the 6-month follow-up versus those who increased their PA to recommended or higher levels over time, and (2) individuals who maintained the recommended PA levels versus those who showed decreases and no longer met the recommended levels at follow-up. We also calculated simple change scores for PA (follow-up minus baseline) to clarify the magnitude of change experienced by each type of user over time. Finally, 2 independent logistic regression analyses were conducted to determine which app features were associated with changes in PA levels. The logistic regression models predicted the 2 binary dependent variables, specifically changes in (and maintenance of) adherence to the recommended PA levels, based on the individual 10 app features and functions used at baseline. All analyses were conducted using R (version 4.2.2; R Foundation) with the following specific packages: chisq.posthoc.test [41], finalfit [42], ggpubr [43], and tidyverse [44].

## Results

### Demographics

Table 1 shows the demographic characteristics for each type of app users (N=16,286). We identified 1462 (8.98%) new users, 2150 (13.20%) continued users, 1899 (11.66%) discontinued users, and 10,775 (66.16%) continued nonusers in the data set. A 1-way analysis of variance indicated significant age differences between the user types (partial η^2^=.009, *P*<.001), implying that new users were older than continued users and continued nonusers, and discontinued users were the youngest among the 4 types of users (*P*<.001; adjusted by the Tukey method). The descriptives per age group showed that, among older participants (eg, ≥60 years) who were identified as app users at baseline, 899 continued app use to follow-up, whereas 675 discontinued their use. Younger app users at baseline (eg, <30 years) were, however, more likely to discontinue than continue app use (n=250 vs 203). Chi-square tests revealed overall significant gender differences (*P*<.001). Residual analyses detected significant gender differences (*P*<.001) within continued users, men (1295/2150, 60.23%) versus women (855/2150, 39.77%). However, within continued nonusers, women (5770/10,775, 53.55%) were more dominant than men (5005/10,775, 46.45%; *P*<.001). Furthermore, continued users were the most prevalent among individuals with the highest household income (≥10 million JPY; 1 JPY=US $0.0065) and education level (university or above). At baseline, most continued users (1609/2150, 74.84%) reported that they had been using an app for longer than 6 months (1128/1899, 59.39%, for discontinued users). Similarly, most continued users (1654/2150, 76.93%) reported using a PA app once or more each day (1153/1899, 60.71%, for discontinued users).

**Table 1:** Demographic statistics of app users and nonusers.

Variable		Current app user		Current nonuser			Total (N=16,286)		*F* test or chi-square (*df*), *P* value
		Continued user (user at baseline; n=2150)	New user (nonuser at baseline; n=1462)	Discontinued user (user at baseline; n=1899)	Continued nonuser (nonuser at baseline; n=10,775)				
Age (years), mean (SD)		53.8 (16.3)	57.3 (16.8)	50.9 (17.3)	55.2 (16.7)	54.7 (16.8)		50.16^a^ (3, 16,282), <.001	
**Age (years; categorical), n (%)**								161.86 (9), <.001	
	<30	203 (9.44)	126 (8.62)	250 (13.16)	965 (8.96)	1544 (9.48)			
	30-44	452 (21.02)	226 (15.46)	502 (26.43)	2093 (19.42)	3273 (20.10)			
	45-59	596 (27.72)	333 (22.78)	472 (24.86)	2859 (26.53)	4260 (26.16)			
	≥60	899 (41.81)	777 (53.15)	675 (35.55)	4858 (45.09)	7209 (44.27)			
Women, n (%)		855 (39.77)	749 (51.23)	915 (48.18)	5770 (53.55)	8289 (50.90)		142.56 (3), <.001	
BMI, mean (SD)		22.4 (3.5)	22.3 (3.9)	22.3 (3.8)	22.0 (3.7)	22.1 (3.7)		7.27^a^ (3, 16,282), <.001	
Married, n (%)		1497 (69.63)	964 (65.94)	1213 (63.88)	6846 (63.54)	10,520 (64.60)		30.68 (3), <.001	
Child/children^b^, n (%)		1412 (65.67)	984 (67.31)	1211 (63.77)	6782 (62.94)	10,389 (63.79)		14.48 (3), .002	
**Education level, n (%)**								231.79 (12), <.001	
	Middle school	27 (1.26)	36 (2.46)	38 (2.00)	290 (2.69)	391 (2.40)			
	High school	517 (24.05)	453 (30.98)	531 (27.96)	3546 (32.91)	5047 (30.99)			
	College or vocational school	382 (17.77)	287 (19.63)	441 (23.22)	2572 (23.87)	3682 (22.61)			
	University or above	1210 (56.28)	673 (46.03)	864 (45.50)	4293 (39.84)	7040 (43.23)			
	Other	14 (0.65)	13 (0.89)	25 (1.32)	74 (0.69)	126 (0.77)			
Job, n (%)		27 (1.26)	36 (2.46)	38 (2.00)	290 (2.69)	391 (2.40)		146.85 (3), (<.001)	
**Household income, n (%)**								335.43 (15), (<.001)	
	<3 million JPY^c^	322 (14.98)	320 (21.89)	416 (21.91)	2487 (23.08)	3545 (21.77)			
	3-5 million JPY	496 (23.07)	350 (23.94)	455 (23.96)	2656 (24.65)	3957 (24.30)			
	5-7 million JPY	365 (16.98)	228 (15.60)	295 (15.53)	1550 (14.39)	2438 (14.97)			
	7-10 million JPY	360 (16.74)	179 (12.24)	236 (12.43)	1135 (10.53)	1910 (11.73)			
	≥10 million JPY	314 (14.60)	115 (7.87)	183 (9.64)	701 (6.51)	1313 (8.06)			
	No response	293 (13.63)	270 (18.47)	314 (16.54)	2246 (20.84)	3123 (19.18)			
Physical activity (baseline, MET hours/week), median (IQR)		34.6 (16.5-67.1)	23.3 (9.9-49.6)	23.3 (7.7-53.5)	11.6 (0.6-33.0)	16.5 (3.3-41.7)		1239 (3), <.001	
Physical activity (follow-up, MET hours/week^d^), median (IQR)		34.0 (16.2-65.2)	24.2 (11.6-51.7)	16.5 (2.5-43.1)	9.9 (0.0-29.7)	14.8 (1.1-38.1)		1419.6 (3), <.001	

^a^*F* test.

^b^Data are from a follow-up survey.

^c^1 JPY=US $0.0065.

^d^Metabolic equivalent task hours per week.

### Relationship Between App Use and Adherence to the Recommended PA Level

Table 2 illustrates changes in PA levels for each user type over 6 months. Continued nonusers generally maintained underrecommended levels of PA over time (5259/10,775, 48.81%). Among the 4 types of users, new users had the largest proportion of individuals who increased their PA to recommended levels (178/1462, 12.18%), although achieving these levels was uncommon in the current sample. Continued users typically maintained the recommended PA levels over time (1327/2150, 61.72%), while discontinued users had the largest proportion of individuals who failed to adhere to the recommended levels at follow-up (330/1899, 17.38%).

**Table 2:** The number of participants who increased, decreased, or maintained physical activity over 6 months as a function of the app user types.a

PA change category (baseline → follow-up)	Current user		Current nonuser	
	Continued user (n=2150), n (%)	New user (n=1462), n (%)	Discontinued user (n=1899), n (%)	Continued nonuser (n=10,775), n (%)
Maintained underrecommended level (not adhered → not adhered)	390 (18.14)	370 (25.31)	638 (33.60)	5259 (48.81)
Increased (not adhered → adhered)	211 (9.81)	178 (12.18)	159 (8.37)	931 (8.64)
Decreased (adhered → not adhered)	222 (10.33)	144 (9.85)	330 (17.38)	1269 (11.78)
Maintained recommended level (adhered → adhered)	1327 (61.72)	770 (52.67)	772 (40.65)	3316 (30.77)

^a^Adherence to the recommended physical activity level is equal to or larger than 23 metabolic equivalent task hours per week for adults or 10 metabolic equivalent task hours per week for older adults aged ≥65 years.

Logistic regression analyses were performed to examine how the 4 types of users were associated with adherence to the recommended PA levels (23 or 10 MET hours/week) over 6 months (primary objective). Discontinued users were used as the reference group, as they showed the largest proportion of individuals who decreased their PA to underrecommended levels at follow-up. Results (Table 3) showed that, compared with discontinued users, continued users (odds ratio [OR] 2.171, 95% CI 1.71-2.76, *P*<.001) and new users (OR 1.93, 95% CI 1.50-2.48, *P*<.001) were more likely to increase PA to the recommended levels at follow-up. Continued nonusers, compared with discontinued users, were more likely to maintain underrecommended PA levels at follow-up (OR 0.71, 95% CI 0.59-0.86, *P*<.001). Another logistic regression analysis showed that continued users (OR 2.56, 95% CI 2.11-3.10, *P*<.001) and new users (OR 2.29, 95% CI 1.84-2.85, *P*<.001) were more likely to maintain the recommended PA levels compared with discontinued users. Continued nonusers did not significantly differ from discontinued users (OR 1.12, 95% CI 0.97-1.29, *P*=.11).

**Table 3:** Logistic regressions predicting physical activity changes based on 23 (or 10) metabolic equivalent task hours per week.

Outcome and predictor			Odds ratio (95% CI)		*P* value
**Increased to versus maintained at below the recommended level (n=8136)**					
	Intercept (reference: discontinued user)	0.249 (0.209-0.297)		<.001	
	Continued user	2.171 (1.705-2.763)		<.001	
	New user	1.930 (1.504-2.477)		<.001	
	Continued nonuser	0.710 (0.589-0.857)		<.001	
**Maintained the recommended level versus decreased (n=8150)**					
	Intercept (reference: discontinued user)	2.339 (2.056-2.661)		<.001	
	Continued user	2.555 (2.109-3.096)		<.001	
	New user	2.286 (1.835-2.847)		<.001	
	Continued nonuser	1.117 (0.967-1.290)		.133	

### Changes in PA Level at the 6-Month Follow-Up

**Figure 2:**
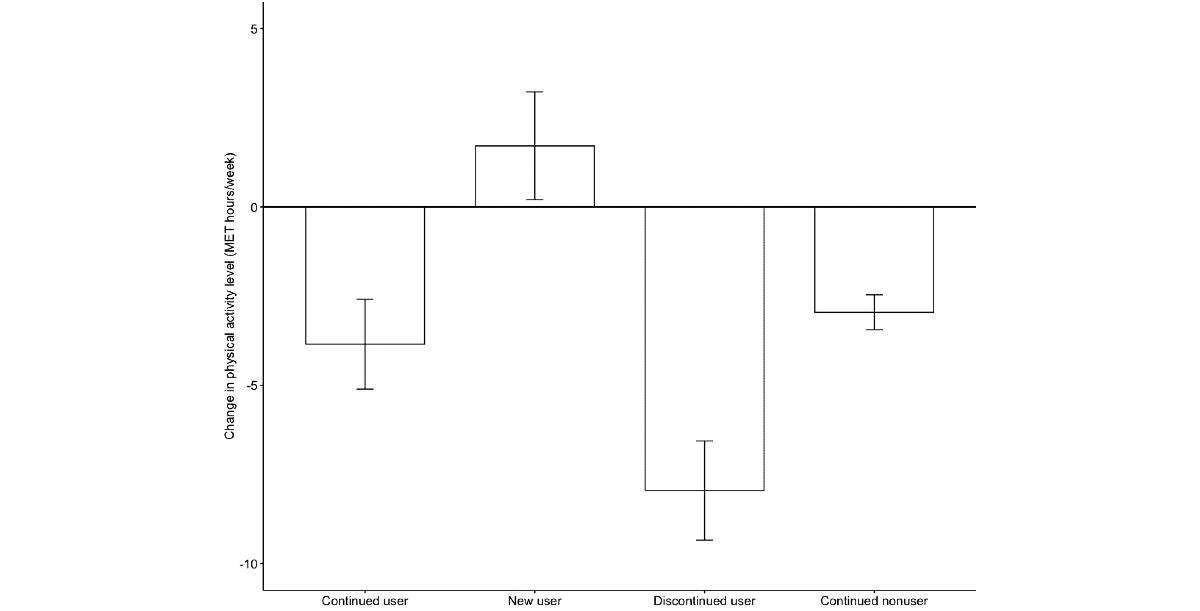
Change in physical activity level (in METs hours/week) at the 6-month follow-up among the 4 types of users and nonusers. The error bar indicates the SE. MET: metabolic equivalent of tasks.

We then calculated the simple change scores for PA levels (ie, follow-up minus baseline in MET hours/week) to estimate the changes experienced by each user type over 6 months (Figure 2). New users were the only group to show increases in PA levels (mean 1.71, SD 57.76), which was significantly larger than the changes (decreases) observed in continued nonusers (mean –2.95, SD 50.76; *t*_1,780.5_= 2.94, *P*=.003). Continued users showed a decrease in PA on average (mean –3.85, SD 58.53), while discontinued users exhibited even larger decreases (mean –7.95, SD 60.52; *t*_3,949.1_=2.19, *P*=.03).

### Function-Wise Analyses Predicting Changes in PA

At baseline, app users most frequently reported using sensor information, followed by goal setting, goal progress, energy analysis, and weight recording (see Table S1 in Multimedia Appendix 1 for details). To explore which app functions are associated with increases in or maintenance of PA levels (secondary objective), we estimated 2 logistic regression models. These analyses targeted continued users exclusively (ie, individuals who reported using apps at both baseline and follow-up), with nonusers excluded. The results (Table 4) showed that energy analysis and journaling were significantly associated with increases in PA to the recommended levels (OR 1.67, 95% CI 1.05-2.64, *P*=.03 and OR 1.76, 95% CI 1.12-2.76, *P*=.01, respectively). Maintenance of the recommended levels (vs a decrease to underrecommended levels) was predicted by goal setting (OR 1.73, 95% CI 1.21-2.48, *P*=.003), sleep information (OR 1.66, 95% CI 1.03-2.68, *P*=.04), and blood pressure recording (OR 2.05, 95% CI 1.10-3.83, *P*=.02). We also tested the association between the number of app functions in use (at baseline) and changes in PA level (simple change score, ie, follow-up minus baseline) among continued users, which did not reach statistical significance (*r*=–0.02, *P*=.48).

**Table 4:** Logistic regression predicting physical activity change based on 23 metabolic equivalent task hours per week.

Outcome and predictor		OR (95% CI)	*P* value
**Increased to versus maintained at below the recommended level (n=601)**			
	Show sensor info	1.008 (0.694-1.464)	.97
	Goal setting	1.005 (0.649-1.557)	.98
	Show goal progress	1.243 (0.789-1.957)	.35
	Energy analysis	1.665 (1.051-2.637)	.03
	Weight recording	0.785 (0.474-1.299)	.34
	Journaling	1.755 (1.116-2.760)	.01
	Global positioning system/maps	0.922 (0.567-1.500)	.74
	Show sleep info	0.991 (0.624-1.573)	.97
	Reward points	1.350 (0.790-2.308)	.27
	Blood pressure recording	1.574 (0.795-3.117)	.19
**Maintained the recommended level versus decreased (n=1549)**			
	Show sensor info	1.200 (0.878-1.640)	.25
	Goal setting	1.729 (1.206-2.480)	.003
	Show goal progress	1.307 (0.900-1.897)	.16
	Energy analysis	0.975 (0.672-1.416)	.90
	Weight recording	1.164 (0.766-1.770)	.48
	Journaling	1.086 (0.748-1.575)	.66
	Global positioning system/maps	1.484 (0.963-2.287)	.07
	Show sleep info	1.658 (1.025-2.684)	.04
	Reward points	1.127 (0.701-1.812)	.62
	Blood pressure recording	2.046 (1.095-3.825)	.02

## Discussion

### Principal Findings

This study investigated whether continued use of commercial PA apps and wearables over 6 months contributes to increasing or maintaining PA levels. Overall, the results emphasize the importance of continued app use in maintaining PA levels. Most continued users (1538/2150, 71.53%) either maintained or improved their PA to the recommended levels over 6 months, whereas 51% (968/1899) of discontinued users failed to meet the recommended levels at follow-up (compared with 797/1899, 41.96%, who were nonadherent at baseline). New users were found to experience the largest increase in PA levels, while discontinued users showed the largest reduction among the 4 types of users. These results suggest that individuals who recently started using apps and wearables saw the greatest improvement in their PA levels. By contrast, continued app use helped maintain PA levels (despite slight decreases), whereas discontinuation led to a substantial reduction in PA levels, equivalent to a decrease of more than 1 hour of vigorous PA per week [45].

### Characteristics of Continued Versus Discontinued Users

More than half (2150/4049, 53.09%) of the app users identified at baseline reported continuing to use the app at the 6-month follow-up. While this retention rate may seem high compared with the reported daily engagement rates for health care apps [30], it is comparable to the 60% active user engagement rate observed in a longitudinal study of a commercial PA app over 6 months [31]. Our analysis of the demographic characteristics of continued (vs discontinued) users indicated that continued users were older, more likely to be men, and had higher education levels and incomes. Previous cross-sectional studies have found that mHealth/eHealth users tend to be younger, more educated, and have higher (digital) health literacy than nonusers [46-48]. Educational attainment is thought to reflect both literacy and skills (including confidence with digital and smart devices) as well as social norms related to the perceived value of health [47]. In general, women are the dominant users of health care apps (for diet, nutrition, and self-care), while fitness apps tend to be more popular among men [46]. Older adults typically avoid new technologies and mHealth services [49]. However, our results showed that continued users were more prevalent than discontinued users among older participants, suggesting that older users were more likely to continue using apps. We do not have data to readily explain this unexpected finding. However, given that most of the continued users in our data had already been using PA apps for a long time (>6 months) at baseline, even older users may have developed high self-efficacy and perceived ease of use, which could reduce technology anxiety. Studies have identified various facilitators and barriers to technology adoption among older adults (eg, personal experiences and subjective norms) [50], which could serve as a basis for future research to explore how older users successfully adapt and integrate mHealth tools into their daily routines.

### App Functions Predictive of an Increase in PA

We found that energy analysis and journaling were predictive of increases in PA to the recommended levels over 6 months. Additionally, goal setting, sleep information, and blood pressure recording were commonly used by individuals who maintained the recommended PA levels at follow-up. In a previous cross-sectional analysis, we reported associations between PA levels and individual app functions [33], which indicated that individuals with health-enhancing PA levels typically used functions such as sensor information (eg, step count and heart rate), goal setting, goal progress, energy analysis, journaling, and GPS/maps. The current prospective analyses emphasize the particular importance of energy analysis and journaling in improving PA over time. While sensor information was commonly used by PA app users (Table S1 in Multimedia Appendix 1), the findings suggest that, in addition to the automatically recorded PA data (eg, step count), users may benefit from additional analyses of physiological data (eg, energy expenditure calculations) and more deliberate engagement with the app, such as journaling and manually logging daily exercise and PA.

Interestingly, maintenance of the recommended PA level was associated with the use of sleep information, blood pressure recording, and goal setting. This suggests that users who are already sufficiently active may value functions that support general health care, rather than those focused solely on fitness and exercise. It is also possible that some users were prompted to use PA apps due to specific health concerns. Published meta-analytic studies have identified key app components that enhance PA, including self-monitoring, goal setting and planning, prompts/cues, feedback on behavior, and action planning [24-27], most of which are specifically designed to support PA. It may be important for future research to broadly explore app functions and features (not limited to PA-related functions) to identify more effective combinations, especially when the focus is on maintaining rather than increasing PA levels. This could reinforce the usefulness of the stages of change model [39,51] (eg, to guide the best interventions for those in the action or maintenance stages) and highlight the importance of tailoring digital behavior interventions.

Another interesting finding from this analysis is that the number of app functions reported as being in use was not significantly associated with increases in PA over 6 months. While several studies have suggested that the amount of app content or the number of implemented BCTs is linked to the efficacy of mHealth interventions [18,19], this association has not always been replicated [29]. As our analyses utilized self-report data rather than actual logs of user behavior, we cannot exclude the possibility that participants may not have accurately or exhaustively reported all the functions they used. However, our findings suggest that (1) users may not be fully aware of every function available in an app (or, at least, they do not consciously use them all), and (2) they may not necessarily benefit from multifunctionality. Instead, a limited number of functions (eg, goal setting and journaling) may be more effective in improving PA. Indeed, it is known that users appreciate the simplicity of an app [20], and as Michie et al [32] found, interventions that combine self-monitoring with at least one regulatory technique (eg, goal setting) can form the most cost-effective, minimal set of interventions.

### Limitations

The results reported here should be interpreted with caution due to several important limitations. First, we targeted Japanese-speaking adults exclusively, which may limit the generalizability of the findings. The apps and products available on the Japanese eHealth/mHealth market may differ from those in other regions and countries. While there are similarities in user behavior between Japan and Western countries, exploring country- or culture-specific aspects would be an interesting direction for future research. Second, we cannot rule out the possibility of sampling bias. As reported elsewhere [33], the current sample exhibited higher PA levels than the general population in Japan, likely because the study was advertised as a survey on PA and health. Additionally, attrition could introduce bias, as some participants (4287/20,573, 20.83%) dropped out by the follow-up. Third, the follow-up survey did not include questions regarding how participants used specific app functions and features (assessed only at baseline). As this study was part of a larger project, there was a limit on the length of each survey. Future research should explore how changes in PA influence the use patterns of individual app functions, which could provide insights into how tailoring and personalization can be incorporated during app use adaptation. Fourth, we relied exclusively on self-reported PA, which may not always align with objective measures, such as accelerometers, due to self-reporting bias and other assessment artifacts. Similarly, user behavior and individual app function usage can be monitored automatically or made publicly available (eg, [52]). However, a downside of such an approach is that the analysis would be limited to a specific app or platform, potentially sacrificing the generalizability of the results. Finally, we cannot rule out the possibility of selection bias. As the surveys were administered online, participants were likely familiar with the internet and possibly mobile technology as well. This could explain the unexpected finding that older individuals were more likely to continue using the app.

### Conclusions

This study demonstrated that continued use of apps and wearables contributes to both increasing and maintaining PA levels over 6 months. The results also revealed that app features associated with increases in PA differ from those linked to the maintenance of PA, highlighting the importance of tailoring apps to users’ PA levels and readiness. We believe these findings make a meaningful contribution to the literature by highlighting the continued use of apps and wearables as key factors in enhancing and maintaining high levels of PA. Additionally, it is noteworthy that more than half of the users continued using the apps through the 6-month follow-up, despite the poor retention rates and barriers commonly reported in the literature (eg, [53,54]). This may suggest that attitudes toward and the acceptability of mHealth apps are changing, with the COVID-19 pandemic potentially serving as an opportunity. Health care practitioners could increasingly rely on app-based approaches in their intervention repertoires, although integrating mHealth into routine practice remains a challenge [55]. Unfortunately, we did not assess the barriers preventing users from continuous engagement (eg, [56,57]), and these should be explored in future research to identify effective strategies for maintaining active user engagement.

## Appendix

Multimedia Appendix 1 Changes in physical activity level among temporary users and use of individual app functions.

## Abbreviations

BCT: behavior change technique
GPS: global positioning system
MET: metabolic equivalent task
mHealth: mobile health
OR: odds ratio
PA: physical activity
