# Supplementary material for: Functions of Smartphone Apps and Wearable Devices Promoting Physical Activity: Six-Month Longitudinal Study on Japanese-Speaking Adults
Source: JMIR Mhealth Uhealth. 2024 Dec 10;12:e59708. doi: 10.2196/59708 (PMC11668998; doi:10.2196/59708)
Supplement: Multimedia Appendix 1 [file mhealth_v12i1e59708_app1.docx]

What Functions of Smartphone Apps and Wearable Devices Promote Physical Activity? Six-Month Longitudinal Study on Japanese-Speaking Adults

Supplemental Materials

Naoki Konishi, Takeyuki Oba, Keisuke Takano, Kentaro Katahira, and Kenta Kimura

Human Informatics and Interaction Research Institute, AIST, Japan

**1. Changes in PA Level among Temporary Users**

Simple change scores of PA (i.e., follow-up minus baseline, in METs-h/w) were calculated for the five types of app users including temporary users (Figure S1). Temporary users showed a non-significant change in PA over six months, M=-1.86, SD=51.11, t(1,850)= 1.06, P=.289. ANOVA revealed that temporary users did not differ from continued nonusers, t(1,987.1) = 0.60, P=.547, but experienced a smaller decrease than discontinued users, t(1,1915.3)=-2.73, P=.007. These results suggest that temporary users could be counted in continued nonusers – these groups did not use apps at all or used apps only to the extent to which PA stayed unchanged. For the main analyses, we decided to leave out temporary users for the simplicity of the analyses as well as for the homogeneity of discontinued nonusers.


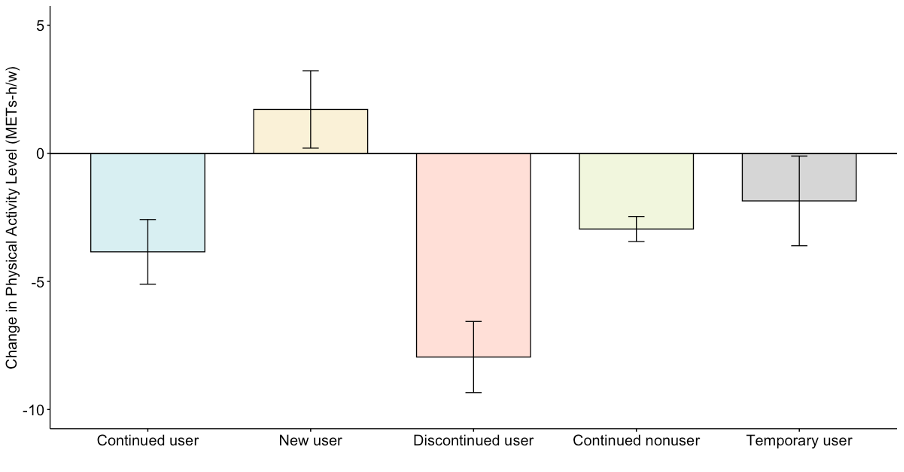


Figure S1. Change in physical activity level (in METs-h/w) over six months for the five types of app users. Error bars indicate the standard error. METs-h/w=metabolic equivalent of tasks, hours per week.

**2. Use of Individual App Functions**

**Table S1:** Number of Users of each App Function among Continued and Discontinued Users

| App function | Continued user, N (%)  N = 2,150 | Discontinued user, N (%)  N = 1,899 |
| --- | --- | --- |
| Show sensor info | 1342 (65.0) | 870 (49.4) |
| Goal setting | 690 (33.4) | 352 (20.0) |
| Show goal progress | 592 (28.7) | 254 (14.4) |
| Energy analysis | 498 (24.1) | 266 (15.1) |
| Weight recording | 481 (23.3) | 255 (14.5) |
| Journaling | 447 (21.7) | 261 (14.8) |
| GPS/map | 406 (19.7) | 244 (13.9) |
| Show sleep info | 397 (19.2) | 164 (9.3) |
| Reward points | 267 (12.9) | 169 (9.6) |
| Blood-pressure recording | 254 (12.3) | 122 (6.9) |

*Note.* Baseline data used.
